# Carrier-free self-assembled nanoparticles derived from traditional Chinese medicine active components: construction strategies and applications

**DOI:** 10.1039/d6ra04326g

**Published:** 2026-08-10

**Authors:** Shuhan Yan, Enyao He, Meiting Chen, Xingbo Wang, Changzhen Sun

**Affiliations:** a Drug Research Center of Integrated Traditional Chinese and Western Medicine, Southwest Medical University Luzhou 646000 China 20220314330218@stu.swmu.edu.cn 20230314331005@stu.swmu.edu.cn 20230314331002@stu.swmu.edu.cn 20230314331001@stu.swmu.edu.cn sunchangzhen@swmu.edu.cn; b Luzhou Key Laboratory of Research and Development of Medical Institution Preparations and Large-Scale Health Products, Southwest Medical University Luzhou 646000 China

## Abstract

Active compounds derived from traditional Chinese medicine (TCM) hold considerable promise for drug development, owing to their natural origin and multi-target synergistic effects. However, their clinical translation has long been limited by poor aqueous solubility, low bioavailability, and inadequate targeting capacity. In recent years, carrier-free self-assembled nanomedicines have emerged as a promising strategy within the field of nano-enabled TCM. Driven by noncovalent interactions, such as hydrophobic forces, hydrogen bonding, and π–π stacking, these systems facilitate the spontaneous assembly of TCM-derived bioactive molecules into well-defined nanostructures. This approach can substantially improve drug loading efficiency, biocompatibility, and therapeutic synergy. This comprehensive review summarizes the underlying assembly mechanisms, key formulation parameters, and recent advances in the application of these nanostructures across antitumor, antibacterial, and anti-inflammatory therapies. It also discusses major challenges hindering large-scale production and clinical translation, including particle size control, stability, safety, and pharmacokinetic behavior. By integrating mechanistic insights with application outcomes and translational considerations, this comprehensive review aims to provide a comprehensive framework for the rational design of self-assembled TCM-based nanomedicines, thereby supporting the modernization of TCM and expanding its potential in innovative drug development.

## Abstract 2

Predictive assembly design, optimized *in vivo* efficacy and standardized evaluation promote steady clinical translation of carrier-free nanomedicines derived from active components of traditional Chinese medicine.

## Graphical abstract

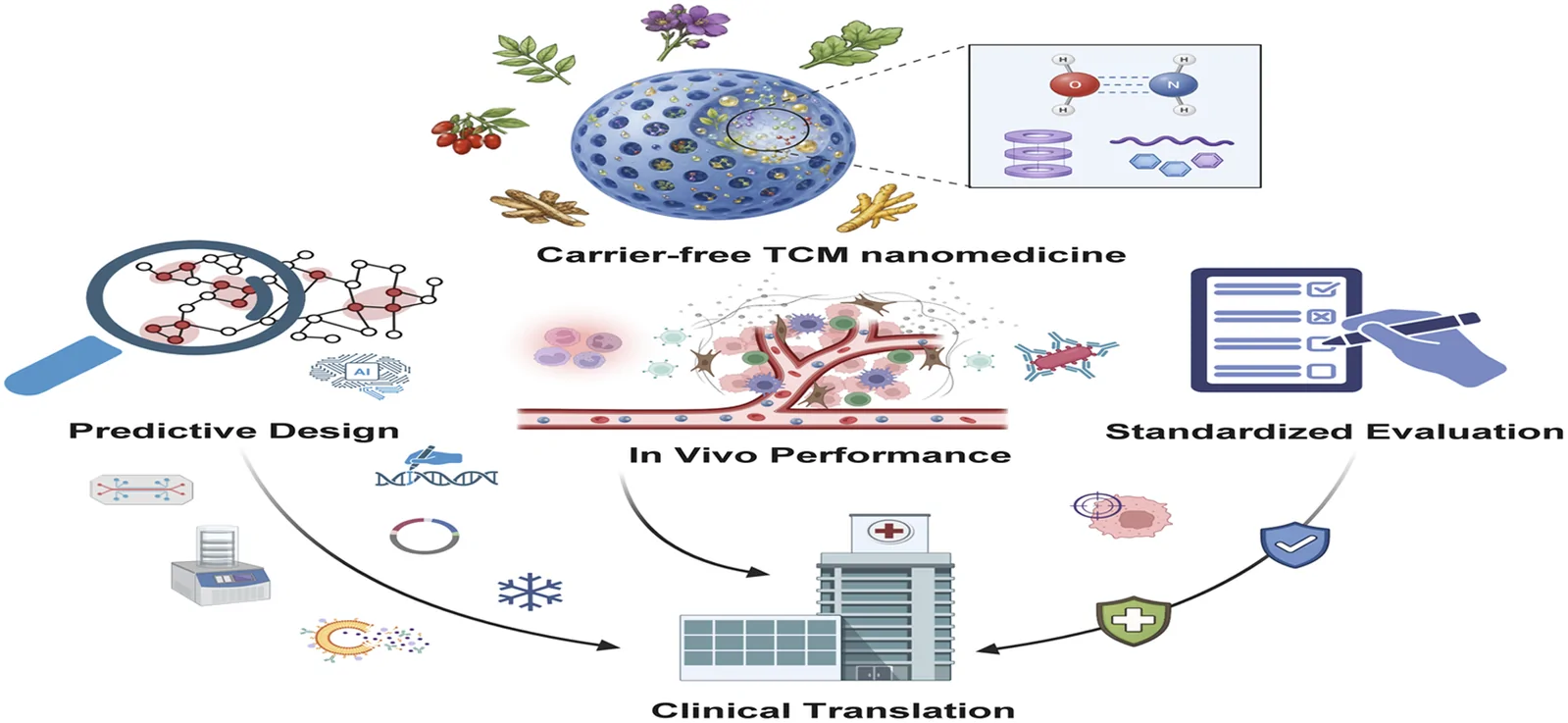

## 1. Introduction

Traditional Chinese Medicine (TCM), developed through thousands of years of clinical practice, remains an integral part of healthcare systems in East Asia and increasingly contributes to the interface between traditional knowledge and modern biomedical science. A growing body of evidence indicates that TCM-derived bioactive compounds possess substantial therapeutic potential for complex diseases, largely due to their multi-target regulatory effects and system-level actions.^1–3^ For instance, berberine (BBR) has shown efficacy in irritable bowel syndrome through coordinated modulation of gut microbiota and immune responses.^4^ Similarly, luteolin, polygonatum polysaccharide, and Mn^2+^ self-assemble into a nanocomplex *via* pH-responsive self-assembly, synergistically inducing oxidative stress, Fenton-like reactions, and anti-tumor immune activation, which has been demonstrated to possess significant therapeutic potential against lung cancer and cervical cancer.^5^

Despite these advances, the clinical translation of TCM active components remains limited by inherent physicochemical constraints. Many compounds exhibit poor aqueous solubility and extremely low bioavailability. For instance, the lipophilic tanshinones from *Salvia miltiorrhiza* (Danshen) and the volatile oil from *Ligusticum chuanxiong* both exhibit extremely poor water solubility and insufficient oral bioavailability, severely limiting the clinical efficacy of the Danshen–Chuanxiong herb pair in treating coronary heart disease,^6^ while berberine and most flavonoids typically show bioavailability below 1% and 5%, respectively.^7,8^ These limitations are further compounded by rapid metabolism and pronounced first-pass effects. Conventional formulation strategies have had limited success in overcoming these barriers and may introduce additional risks, including off-target toxicity arising from non-specific tissue distribution.^9^ In addition, multi-component formulations often face challenges in chemical consistency across different dosage forms. For instance, the contents of amygdalin and magnoloside A in excipient-free extracts are significantly higher than those in commercial granules, whereas sennoside A remains relatively stable. Such formulation-dependent discrepancies constrain the quality controllability and clinical translation of compound traditional Chinese medicine prescriptions.^10^

The application of nanotechnology offers new opportunities to address these challenges. Since the concept of “nano-TCM” was proposed in 1998,^11^ nanocarrier-based delivery systems have improved drug solubility and delivery efficiency to some extent. However, the use of exogenous carrier materials introduces additional safety concerns. For example, solid lipid nanoparticles loaded with podophyllotoxin can induce systemic toxicity at concentrations as low as 50 mg L^−1^,^12^ while long-term accumulation of gold nanoparticles *in vivo* may lead to cellular dysfunction.^13^ Moreover, carrier materials may alter the pharmacokinetics and toxicological profiles of TCM-derived compounds.^14^ Against this backdrop, carrier-free self-assembled nanomedicines have attracted increasing attention. This strategy employs intrinsic intermolecular interactions, such as hydrophobic forces, hydrogen bonding, and π–π stacking, to enable the spontaneous assembly of TCM-derived molecules into nanoscale structures. For instance, the rigid planar structure and charge characteristics of BBR facilitate its self-assembly into stable nanostructures.^15,16^ By eliminating the need for external carriers, this approach reduces carrier-associated risks while enabling high drug loading (up to 93.3%) and relatively simple, environmentally friendly preparation processes.^17,18^

This review summarizes recent progress in carrier-free self-assembled nanomedicines, focusing on their design strategies, construction mechanisms, and therapeutic applications. It also discusses key challenges related to large-scale production and clinical translation, with the aim of providing a theoretical framework to support the modernization of TCM and the development of next-generation therapeutics.

## 2. Carrier-free self-assembled nanodrugs: from molecular insights to rational design

**Fig. 1:**
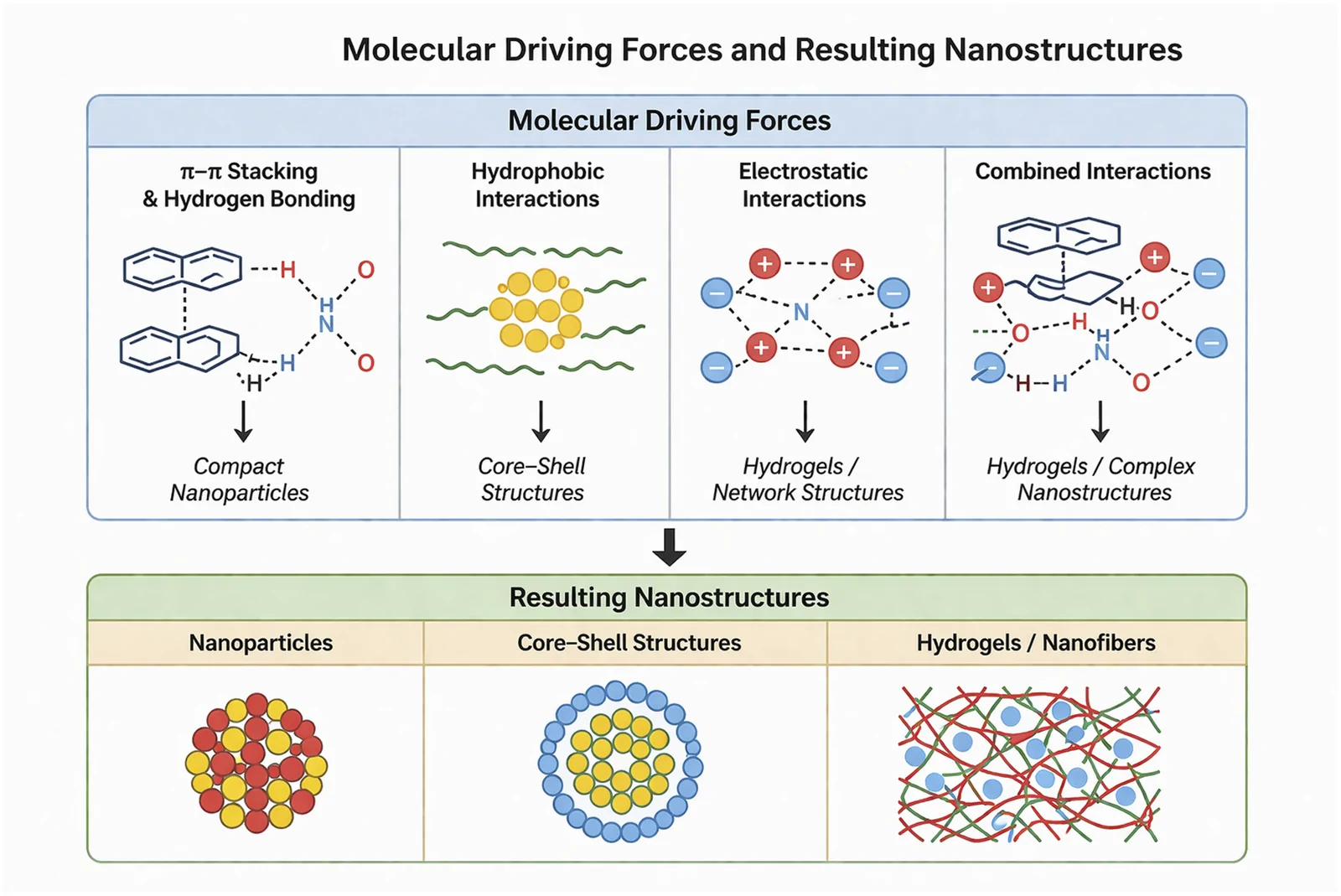
Schematic illustration of molecular driving forces and resulting morphologies in carrier-free TCM nanomedicines.

Carrier-free self-assembled nanomedicines derived from TCM are formed through the spontaneous organization of bioactive molecules driven by noncovalent interactions, including π–π stacking, hydrogen bonding, hydrophobic forces, and electrostatic interactions. Unlike conventional carrier-based systems, these formulations rely on the intrinsic physicochemical properties of the drug molecules to form stable nanoscale architectures with high drug loading (Table 1). These noncovalent interactions not only initiate self-assembly but also determine the resulting nanostructures. Specifically, π–π stacking and hydrogen bonding tend to drive the formation of compact nanoparticles, hydrophobic interactions often promote core–shell architectures, while multivalent electrostatic interactions, alone or in combination with other forces, can give rise to hydrogel or network-like structures (Fig. 1).

**Table 1:** Comparative analysis of representative carrier-free nanomedicines

System type	Particle size	Drug loading	Stability	Therapeutic effect	Key limitations
Drug–drug self-assembly (*e.g.*, BBR-based)	50–200 nm	Very high (>80–90%)	Moderate (aggregation risk)	Strong synergy, antimicrobial/anticancer	Poor stability, batch variability
Covalent conjugate self-assembly	80–150 nm	High (∼70–90%)	Improved *vs.* noncovalent	Controlled release, better PK	Complex synthesis, regulatory challenges
Hybrid (HA-modified, PDA-coated)	100–250 nm	Moderate-high	High	Enhanced targeting, longer circulation	Not strictly carrier-free
Carrier-containing (*e.g.*, ZIF-8, PEG-PCL)	100–300 nm	Moderate (10–50%)	High	Well-controlled delivery	Carrier toxicity, lower drug fraction

Based on composition and assembly strategy, these nanosystems can be categorized into three classes: (i) pure drug–drug self-assembly systems, in which two or more pharmacologically active components spontaneously assemble *via* noncovalent interactions (*e.g.*, π–π stacking, hydrogen bonding, and hydrophobic interactions). (ii) Covalent drug–drug conjugate self-assembly systems, where active molecules are chemically linked to form amphiphilic or structurally programmable units that subsequently undergo self-assembly. Despite covalent modification, the resulting nanostructures remain composed entirely of active pharmaceutical ingredients. (iii) Hybrid or surface-modified self-assembled systems, which consist of a carrier-free core with limited functional modifications, such as targeting ligands, biomimetic coatings, or responsive shells. These systems are considered quasi-carrier-free only when such modifications are minimal and do not dominate the structural framework or mass composition of the nanoparticle. For comparison, carrier-containing nanocomposites are classified separately. In these systems, exogenous materials, including polymer matrices (*e.g.*, PEG-based systems), metal–organic frameworks (*e.g.*, ZIF-8), and lipid-based carriers, constitute a structurally dominant component of the nanoplatform.

By exploiting the intrinsic self-assembly properties of drug molecules, this strategy addresses key limitations of TCM bioactive compounds, such as poor solubility and low bioavailability, while avoiding the potential toxicity associated with conventional nanocarriers. Evidence from *in vitro* and early *in vivo* studies suggests that these “pure drug” nanostructures can significantly enhance therapeutic efficacy, offering a promising direction for advancing TCM-based drug development.

### 2.1 Molecular logic of self-assembly: building blocks and driving force landscapes

#### 2.1.1 Structural repertoire and functional versatility of self-assembling active components

The selection of suitable TCM-derived active components represents the first step in constructing carrier-free self-assembled nanomedicines. Not all natural products possess sufficient self-assembly capability. Successful assembly generally depends on specific structural features, including planar aromatic rings, amphiphilic characteristics, abundant hydrogen-bonding sites, and complementary electrostatic interactions. Based on their chemical structures, alkaloids, flavonoids, terpenoids, and polyphenols constitute the major classes of TCM-derived compounds used for constructing carrier-free self-assembled nanosystems. Among alkaloids, BBR exhibits strong self-assembly capability due to its rigid planar aromatic structure and quaternary ammonium cation. Often regarded as a model compound in this field, BBR can co-assemble with various small molecules, including gallic acid (GA) and rhein (RH). For example, BBR and GA form GA-BBR nanoparticles that inhibit bacterial growth by disrupting protein translation and preventing biofilm formation.^19^ In addition, BBR can assemble with indocyanine green (ICG) to form multifunctional BBR–ICG nanoparticles for the treatment of infected wounds through synergistic effects.^20^ BBR also forms a linear self-assembled complex with aristolochic acid (AA). This process begins with electrostatic interactions between the carboxyl group of AA and the quaternary ammonium group of BBR, followed by stabilization through additional intermolecular forces. Notably, this assembly suppresses the cyclization of AA's toxic functional groups (nitro and hydroxyl), inhibits the formation of its toxic metabolite (aristolochic acid lactam), and reduces DNA adduct formation, thereby significantly mitigating its toxicity.^21^ Triterpenoids such as ursolic acid (UA) can spontaneously self-assemble into stable nanoparticles (approximately 150 nm in diameter) due to their strong hydrophobicity, even as single-component systems. This property makes them suitable as hydrophobic cores for constructing composite nanoparticles, such as UA-paclitaxel (PTX) systems. These formulations can prolong plasma half-life, reduce premature drug leakage, and enable co-delivery while preserving the antitumor and hepatoprotective activities of UA.^22^ More broadly, terpenoids constitute an important source of lead compounds in drug discovery because of their structural diversity and wide-ranging bioactivities, including anti-inflammatory, antibacterial, and antitumor effects.^23^ Baicalin (BA), a representative flavonoid, can spontaneously form BA–BBR complex nanocrystals during co-decoction with BBR. Optimized BA–BBR nanocrystals have been shown to enhance dissolution rate and extent, improve oral bioavailability, and promote co-absorption of both components.^24^ In addition, BBR can co-assemble with chlorogenic acid (CGA) at pH 7.0–7.5 to form BBR–CGA nanoparticles, which exhibit potent antibacterial activity, achieving 99.06% inhibition of methicillin-resistant *Staphylococcus aureus* (MRSA), markedly higher than that of ampicillin (29.03%).^25^

#### 2.1.2 Cooperative noncovalent interaction networks and their physicochemical determinants

Following the identification of suitable TCM-derived components, understanding the molecular mechanisms governing self-assembly is essential for the rational design of stable carrier-free nanomedicines. Unlike conventional nanocarriers, these systems are assembled primarily through cooperative noncovalent interactions, including hydrogen bonding, π–π stacking, hydrophobic interactions, and electrostatic attraction, which collectively determine assembly morphology, stability, and biological properties. Self-assembly is governed by the minimization of Gibbs free energy (Δ*G*), arising from a balance between enthalpic contributions (*e.g.*, hydrogen bonding, π–π stacking, and electrostatic interactions) and entropic gains, particularly those associated with hydrophobic interactions and solvent reorganization (Table 2). Molecular docking studies have shown that BBR can selectively bind to the active-site pocket of bacterial efflux pump proteins (*e.g.*, AcrB) through electrostatic and hydrophobic interactions, forming stable complexes that competitively inhibit antibiotic efflux and restore the susceptibility of multidrug-resistant bacteria to conventional antibiotics.^26^ In another study, the *Scutellaria baicalensis*–*Coptis chinensis* herb pair and its major constituents baicalin–berberine (BA–BBR) can self-assemble into nanofibers (NFs) through simple mechanical mixing, whereas thermal treatment induces a thermodynamically driven supramolecular phase transition, yielding nanospheres (NPs). Specifically, mechanically mixed BA–BBR assembles into nanofibers, while heated BA–BBR undergoes a morphological shift to nanospheres. The nanospheres exhibit enhanced antibacterial activity against *Staphylococcus aureus*, which is mechanistically attributed to the disruption of amino acid biosynthesis and metabolism. In contrast, the inhibitory effect of nanofibers on *Bacillus subtilis* is mediated by physical entanglement and interference with energy metabolism, revealing a distinct antibacterial spectrum compared with that of nanospheres. These findings underscore the critical role of assembly morphology in dictating biological activity.^27^ Structural variations further contribute to functional differences: nanoparticles, with surface-exposed hydrophilic glycosyl groups, demonstrate greater bacterial affinity and antimicrobial efficacy, whereas nanofibers exhibit comparatively reduced activity due to constraints imposed by their spatial configuration. Importantly, both studies report favorable biocompatibility, highlighting the value of noncovalent synergistic interactions in the design of efficient and low-toxicity TCM-based antimicrobial nanomaterials.

**Table 2:** Mechanisms underlying the formation of self-assembled nanostructures from active components of traditional Chinese medicine

Classification	Key components	Source plant	Co-assembling partner	Assembly driving forces	Structural outcome	Functional outcome/Target	Ref.
Alkaloids	Berberine (BBR)	*Coptis chinensis*, *Phellodendron amurense*, *etc.*	Gallic acid (GA)	π–π stacking, hydrogen bonding	Nanoparticles	Inhibits bacterial growth and biofilm formation	19
			Indocyanine green (ICG)	Hydrophobic interaction, π–π stacking	Hybrid nanoparticles	Photothermal + antimicrobial synergy	20
			Aristolochic acid (AA)	Electrostatic interaction, hydrogen bonding	Linear complexes	Reduced AA toxicity and DNA adduct formation	21
			Baicalin (BA)	Electrostatic interaction, hydrophobic forces	Nanocrystals	Enhanced dissolution and oral bioavailability	24
			Chlorogenic acid (CGA)	Hydrogen bonding, π–π stacking	Nanoparticles	Strong antibacterial activity (*e.g.*, MRSA inhibition)	25
				Electrostatic and hydrophobic interactions		Binds bacterial efflux pump proteins, competitively inhibits antibiotic efflux, and reverses bacterial resistance	26
Triterpenes	Ursolic acid (UA)	*Ligustrum lucidum*, *Gardenia jasminoides*, *etc.*	Paclitaxel (PTX)	Hydrophobic interaction	Core-shell nanoparticles	Prolonged circulation and synergistic antitumor effect	22
Flavonoids	Baicalin (BA)	*Scutellaria baicalensis*	Berberine (BBR)	Electrostatic and hydrophobic interaction	Nanoparticles	Enchanced antibacterial activity	27
	Baicalein (BAI)			Hydrophobic interaction	Nanofibers	Reduced antibacterial activity due to morphology	28

Beyond molecular interactions, physicochemical parameters play a critical role in determining the stability and biological performance of these systems. Key attributes include particle size and size distribution, polydispersity index (PDI), and zeta potential, which collectively influence colloidal stability, aggregation behavior, and circulation dynamics. For example, nanoparticles with low PDI values (<0.2) typically exhibit improved uniformity and reproducibility, while surface charge (zeta potential) affects electrostatic stabilization and interactions with biological membranes. In physiological environments, stability is further challenged by the presence of serum proteins, which can adsorb onto nanoparticle surfaces to form a protein corona, thereby altering particle size, surface properties, and cellular uptake behavior. Advanced characterization techniques are essential to elucidate these properties and underlying mechanisms. Cryogenic transmission electron microscopy (cryo-TEM) enables visualization of nanostructures in their native hydrated state, while small-angle X-ray scattering (SAXS) provides insights into internal structural organization and assembly patterns. Isothermal titration calorimetry (ITC) further allows quantitative analysis of binding affinities and thermodynamic parameters governing molecular interactions. Importantly, the *in vivo* stability of carrier-free self-assembled systems remains a key challenge. These nanostructures may undergo partial or complete disassembly upon dilution in plasma, competitive binding with serum proteins, enzymatic degradation, or mechanical stress during circulation. Such destabilization can affect drug release profiles, targeting efficiency, and therapeutic outcomes.

Although these strategies demonstrate significant advantages in improving self-assembly efficiency and structural control, they are often sensitive to process parameters and may introduce challenges in reproducibility and large-scale manufacturing, which are discussed in detail in Section 4.1.1.

### 2.2 Controlled construction of self-assembled nanodrugs: from methodologies to strategic advances

**Fig. 2:**
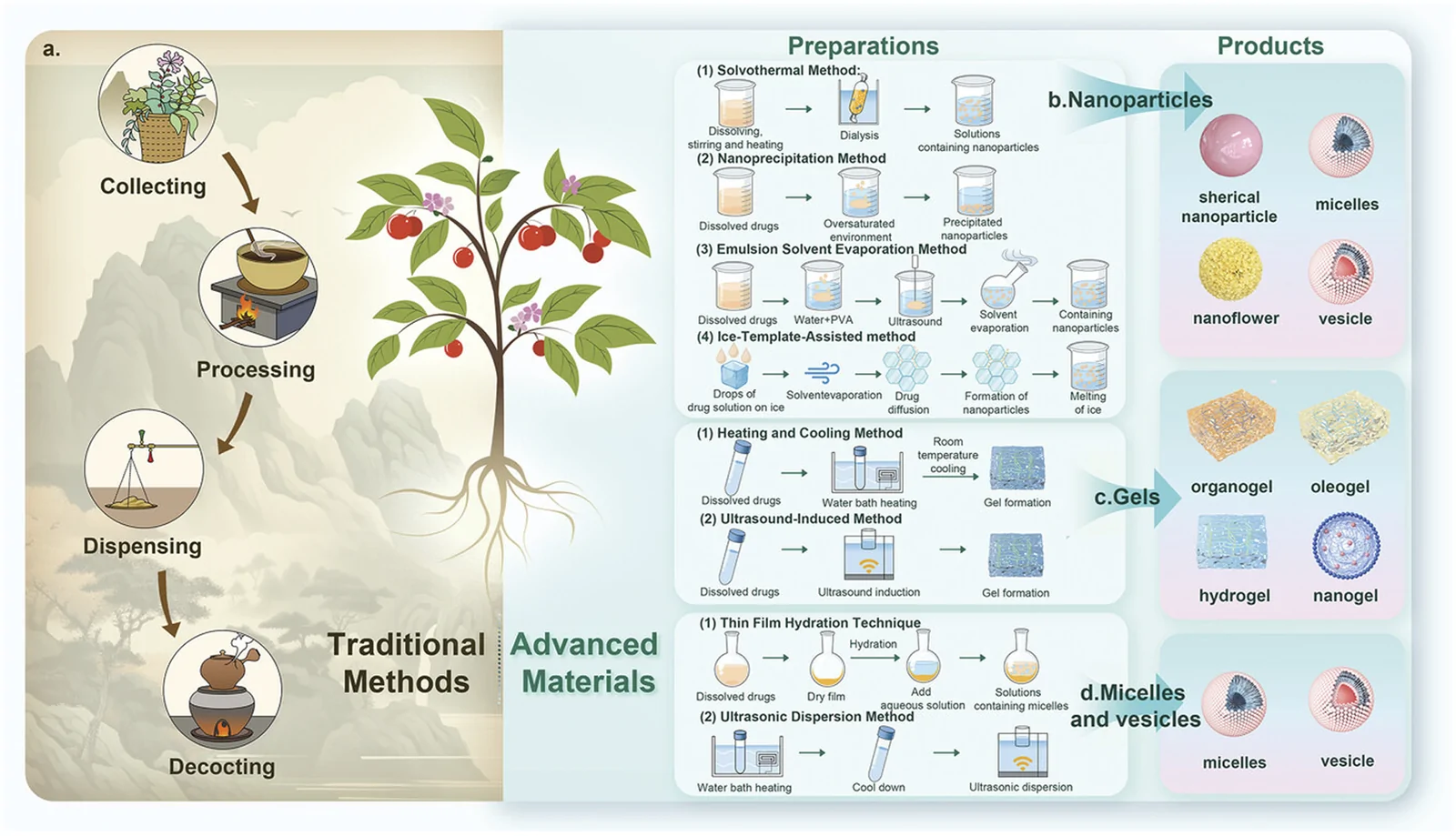
Evolution of human utilization of herbs: from traditional methods to advanced techniques and materials. (a) Traditional steps in the utilization of herbs medicines for the treatment of diseases. (b) Preparations of NPHM-based supramolecular nanoparticles. (c) Preparations of NPHM-based supramolecular gels. (d) Preparation of NPHM-based supramolecular gels. Reproduced from ref. 28 with permission from Wiley, copyright 2024.

Once suitable components and their self-assembly mechanisms have been identified, appropriate preparation strategies are required to control nanoparticle formation, morphology, and physicochemical properties. Because TCM-derived compounds differ substantially in hydrophobicity, crystallization behavior, and hydrogen-bonding capacity, multiple carrier-free self-assembly approaches have been developed to achieve efficient fabrication and precise control of nanomedicines (Fig. 2). Representative preparation methods include solvent evaporation, ice templating, environment-responsive assembly, and biomimetic modification, each offering distinct advantages depending on the physicochemical characteristics of the active compounds. These preparation strategies generally involve three sequential steps: (i) dissolution or molecular dispersion of the active compounds in an appropriate solvent system, (ii) induction of molecular self-assembly through controlled changes in the surrounding environment, and (iii) stabilization and recovery of the resulting nanostructures. The principal distinction among these methods lies in the mechanism used to initiate molecular assembly. In the solvent evaporation method, gradual removal of a volatile organic solvent increases molecular supersaturation, thereby promoting spontaneous self-assembly through hydrophobic interactions, hydrogen bonding, and π–π stacking. This approach is particularly suitable for hydrophobic compounds with strong self-association tendencies. In contrast, ice-templating employs directional ice crystal growth during freezing to spatially concentrate drug molecules within the unfrozen regions, guiding their ordered assembly. Subsequent freeze–drying or freeze–thaw processing removes the ice template while preserving the assembled nanostructure. Compared with conventional solvent evaporation, ice templating is a surfactant-free strategy that offers higher production yield and improved control over particle size and morphology, making it especially attractive for poorly water-soluble compounds.^28^ For example, gallic acid dimer (GBA_2_), owing to its strong hydrophobicity and self-association properties, can be induced to form uniformly sized spherical nanoparticles *via* solvent evaporation, achieving drug loading efficiencies exceeding 90%. These nanoparticles exhibit effective targeting of joint inflammation and demonstrate therapeutic potential in rheumatoid arthritis (RA). Molecular dynamics simulations further indicate that the assembly process is primarily driven by hydrophobic interactions and π–π stacking.^29^

#### 2.2.1 Sustainable preparation technologies toward green manufacturing

In terms of green and sustainable preparation technologies, ice templating has introduced a scalable approach for producing nanomedicines from hydrophobic TCM-derived compounds. Using curcumin as a model system, the ice-template-assisted method directs drug molecule assembly through ice crystal growth, producing nanoparticles *via* freeze–thaw or freeze–drying cycles. In the original study by Zhang *et al.*,^30^ the yield of curcumin pure nanodrugs (PNDs) prepared using this approach was reported to be approximately 140-fold higher than that obtained *via* conventional reprecipitation under comparable experimental conditions, without the use of surfactants or carriers. Furthermore, particle sizes could be tuned within the ∼20–200 nm range by adjusting processing parameters, demonstrating the method's controllability and potential scalability for hydrophobic drug systems. To achieve synergistic therapeutic effects, drug–drug conjugation-guided self-assembly has also emerged as a promising strategy. For instance, covalent conjugation of curcumin with erlotinib enables the spontaneous formation of stable nanostructures. These assemblies not only enhance cytotoxicity, and inhibit migration and invasion of pancreatic cancer cells, but also improve tumor targeting by exploiting the enhanced permeability and retention (EPR) effect, ultimately prolonging survival in tumor-bearing mice.^31^

#### 2.2.2 Pathological microenvironment-inspired stimuli-responsive preparation strategies

Environment-responsive strategies enable precise control over nanostructure formation by modulating external conditions. pH- and temperature-triggered approaches can induce conformational changes that drive self-assembly; for example, BA and CGA spontaneously assemble into stable nanostructures at pH 7.0–7.5.^32^ Counter-solvent precipitation provides another route for controlling nanoparticle size and morphology by introducing a poorly miscible solvent, thereby inducing rapid nucleation and growth.^33^ For highly crystalline and poorly water-soluble anticancer drugs such as lapatinib ditosylate, wet media milling combined with freeze–drying technology can yield nanocrystalline solid dispersions with markedly enhanced dissolution rates. This approach effectively improves bioavailability while minimizing degradation and polymorphic transformation.^34^

#### 2.2.3 Biomimetic interfacial engineering for active-targeting delivery

Biomimetic modification offers effective strategies to improve the *in vivo* performance of nanomedicines. For example, ginsenoside Rb_1_ (GRb1) and probucol can co-assemble into stable nanoparticles *via* noncovalent interactions. When further encapsulated within macrophage-derived microvesicles, these nanoparticles exhibit enhanced targeting to atherosclerotic plaques, resulting in synergistic antioxidant, anti-inflammatory, and lipid-regulating effects, along with improved safety profiles.^35^ Similarly, nanoparticles coated with erythrocyte membranes demonstrate prolonged circulation times, with half-lives of up to 39.6 hours, while reducing nonspecific accumulation in organs such as the liver and spleen.^36^ Lactoferrin (LF)-modified nanoparticles exploit their affinity for transferrin receptors (TFR) and lactoferrin receptors (LFR) to facilitate transport across the blood–brain barrier or enable targeted uptake by tumor cells.^37^ These findings highlight that optimizing self-assembly strategies according to molecular physicochemical properties, combined with biomimetic surface engineering, represents a key direction for advancing the clinical translation of TCM-based nanomedicines.

### 2.3 Stability engineering of self-assembled nanodrugs: from degradation pathways to reinforcement strategies

The stability of carrier-free self-assembled nanomedicines is governed by the dynamic interplay between intrinsic molecular interactions that maintain supramolecular organization and extrinsic environmental factors that perturb these interactions.^38^ Unlike conventional nanocarriers, which derive structural support from polymeric or lipid matrices, carrier-free systems rely exclusively on reversible noncovalent interactions to preserve their structural integrity. Hence, the stability of these assemblies depends on maintaining a favorable thermodynamic balance between the attractive forces that drive self-assembly and the disruptive forces encountered during storage and biological circulation.

At the molecular level, the stability of carrier-free assemblies is primarily dictated by the physicochemical characteristics of the constituent molecules and their packing arrangements.^39^ Aromatic molecules with extended conjugated structures facilitate strong π–π stacking interactions, thereby lowering the free energy of the assembled state and promoting ordered molecular packing. Hydrogen bonding further reinforces the supramolecular architecture by providing directional and cooperative interactions that restrict molecular mobility and increase structural rigidity. Hydrophobic interactions constitute another major driving force for self-assembly, as the sequestration of hydrophobic moieties from the aqueous environment minimizes interfacial free energy and stabilizes the nanoparticle core. Electrostatic interactions also play a dual role in determining assembly stability. Although an appropriate surface charge generates sufficient electrostatic repulsion to prevent interparticle aggregation and maintain colloidal stability, excessive charge neutralization or ionic shielding can diminish repulsive forces and promote nanoparticle aggregation. Furthermore, the overall assembly architecture, including molecular packing density, crystallinity, particle morphology, and size, determines the kinetic stability of the nanostructure by influencing the energy barrier required for molecular dissociation, structural rearrangement, and drug leakage.

Following systemic administration, carrier-free nanomedicines are exposed to complex physiological environments that continuously challenge their structural integrity by modulating the intermolecular interactions responsible for self-assembly. Variations in pH alter the protonation state of ionizable functional groups, thereby affecting hydrogen bonding, electrostatic interactions, and molecular solubility, which collectively influence the equilibrium between assembled and dissociated states. Likewise, increased ionic strength in biological fluids screens electrostatic interactions through the Debye shielding effect, reducing electrostatic repulsion between nanoparticles and increasing their susceptibility to aggregation. Dilution upon entry into the bloodstream decreases the concentration of free drug molecules, potentially shifting the dynamic equilibrium toward partial disassembly when the concentration falls below that required to sustain supramolecular assembly. In addition, rapid adsorption of plasma proteins onto the nanoparticle surface results in the formation of a protein corona, which modifies surface chemistry by masking functional groups, redistributing surface charge, and altering hydration layers.^40^ These changes may disrupt the intermolecular interactions stabilizing the assembly, facilitate particle aggregation, modify cellular uptake pathways, and accelerate recognition and clearance by the mononuclear phagocyte system. Enzymatic degradation further compromises structural stability by chemically modifying or degrading the constituent molecules, thereby weakening the noncovalent interactions responsible for assembly. Furthermore, mechanical shear stress generated during blood circulation can enhance nanoparticle deformation, molecular exchange, and collision frequency, accelerating structural rearrangement in assemblies stabilized predominantly by relatively weak supramolecular interactions.

The molecular composition, assembly mechanism, preparation strategy, and physicochemical stability of carrier-free nanomedicines collectively determine their biological performance. The following section summarizes recent therapeutic applications of these nanosystems in antibacterial therapy, inflammation, metabolic disorders, cancer, and other diseases.

## 3. Progress in application fields

Building on these design principles, carrier-free self-assembled nanosystems derived from TCM have been increasingly explored across a range of disease models, particularly in antibacterial, anti-inflammatory, and anticancer applications. Their therapeutic performance is closely associated with enhanced solubility, improved cellular uptake, and the ability to achieve multi-component synergistic effects without the need for exogenous carriers. In addition, the absence of inert materials may reduce carrier-associated toxicity while achieving higher drug payloads. This section summarizes representative studies in major application areas, highlighting how specific assembly strategies, physicochemical properties, and targeting mechanisms translate into improved therapeutic outcomes *in vitro* and *in vivo* (Table 3).

**Table 3:** Research progress in application fields of self-assembled nanoformulations based on active components of traditional Chinese medicine

Nanocomposite	Therapeutic applications	Mechanism of action	Ref.
Berberine (BBR) and cinnamic acid (CINN)	MRSA infection, biofilm removal	Self-assembles into nanoparticles *via* hydrogen bonding and π–π stacking, actively adsorbs and penetrates bacterial cells, exerts multiple bactericidal activities, and exhibits multi-pathway synergistic antibacterial effects	41
BBR and rheum acid (RHE)	Inhibits bacteria, reduces minimum inhibitory concentration, and disrupts biofilms	Self-assembles into a layered, space-embedded configuration to disrupt bacterial biofilms and cellular structures, achieving highly effective bactericidal activity	42
Baicalin (BA) and sanguinarine (SAN)	MRSA infection, promotes healing as an injectable wound dressing	Self-assembles into hydrogels *via* electrostatic interactions, π–π stacking, and hydrogen bonding; inhibits expression of bacterial virulence factors, reduces inflammatory responses, and promotes wound healing	43
Traditional Chinese medicine extracts (TCMEs), such as those from honeysuckle and *Astragalus*	*Staphylococcus aureus* and *Pseudomonas aeruginosa* infections	Green synthesis of silver nanoparticles using reducing agents and dispersants, inhibiting bacterial growth and controlling pathogen spread	44
Curcumin and 2-deoxy-d-glucose (2-DG)	Rheumatoid arthritis (RA)	Co-assembled into a carrier-free nanosystem, leveraging 2-DG's affinity for glucose transporters to target inflamed joints, inhibiting glycolysis and synergizing with curcumin's anti-inflammatory effects to regulate metabolism and immunity	51
BBR, tannic acid (TA), and hyaluronic acid (HA)	Ulcerative colitis (UC)	HA-coated BBR–TA self-assembled nanosystems mediate colonic targeting *via* CD44 receptors, enhancing drug accumulation at the lesion site and alleviating inflammation	58
Curcumin hybrid assembly (containing azoreductase-sensitive bond polymer and TPGS)	UC	Utilizes colonic azoreductase-triggered release to modulate gut microbiota and TLR4/MyD88/NF-κB signaling pathways, alleviating colitis symptoms and promoting mucosal repair	60
Ursolic acid (UA) and epigallocatechin gallate (EGCG)	Hepatocellular carcinoma (HCC)	Green co-assembly forms a core–shell nanomaterial system. EpCAM aptamer modification enables active targeting, activating innate and adaptive immunity for synergistic antitumor effects	64
BBR and artemisinin	HCC	Self-assembled nanoparticles without carriers. Co-assembly produces complementary antitumor effects, enhancing cytotoxicity *via* synchronized ROS generation and apoptosis induction	65
Celastrol (CE) and galactose (Gal)	HCC	CE and Gal self-assemble into nanoparticles after conjugation. Gal targets the sialic acid-depleted glycoprotein receptors on HCC cell surfaces, while the EPR effect facilitates tumor accumulation. CE induces ROS generation, downregulates GPX4, and upregulates COX-2, triggering ferroptosis	66
Dihydroartemisinin (DHA) and chlorin e6 (Ce6)	Breast cancer	Encapsulating DHA and Ce6 *via* PEG-PCL carriers enhances drug solubility and tumor targeting, induces ROS generation and apoptosis, and boosts photodynamic therapy efficacy	67
BA	Breast cancer	The nanodelivery system modulates TGF-β signaling, influences lncRNA-MALAT1 expression, upregulates miR-200c, inhibits ZEB1/2 transcription factors, reverses epithelial–mesenchymal transition (EMT), and suppresses cancer cell migration and invasion	69 and 70
BA in folate-modified ZIF-8 nanosystems	Breast cancer	Leveraging the high drug-loading capacity of the metal–organic framework ZIF-8, folate modification enables active targeting, enhancing drug accumulation efficiency at tumor sites and improving antitumor efficacy	71

### 3.1 Antibacterial applications

**Fig. 3:**
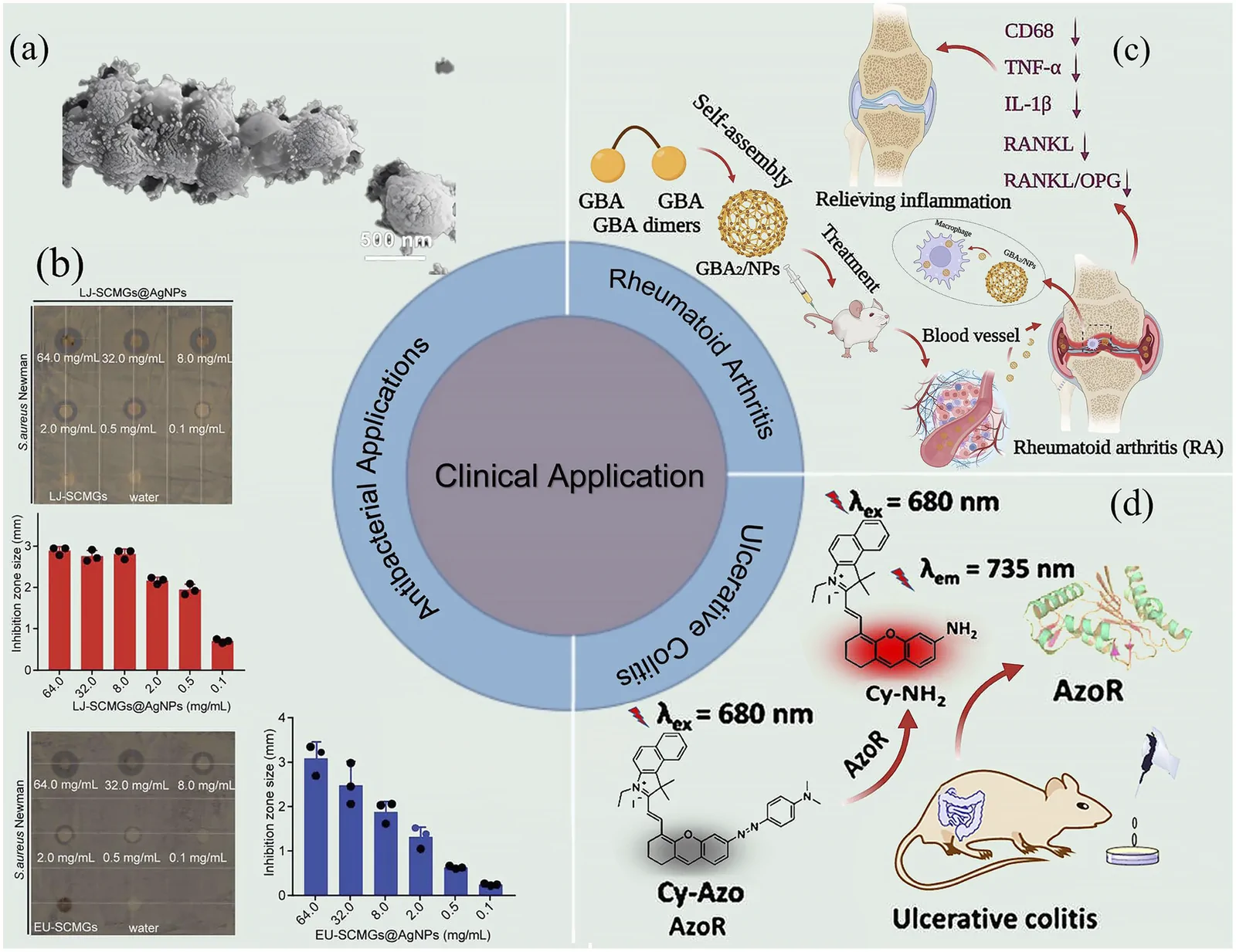
Created with https://www.BioGDP.com (accessed on 10 February 2026). (a) Interaction between CA-BBR nanoparticles and methicillin-resistant *Staphylococcus aureus* (MRSA). Reproduced from ref. 41 with permission from American Chemical Society, copyright 2020. (b) Antibacterial activity of traditional Chinese medicine-chitosan microspheres loaded with silver nanoparticles. Reproduced from ref. 44 with permission from the Royal Society of Chemistry, copyright 2021. (c) Cleavage of specific chemical bonds in nanocarriers or prodrugs by enzymes such as azoreductase. Reproduced from ref. 54 with permission from Dove Medical Press, copyright 2023. (d) Treatment of rheumatoid arthritis *via* dual targeting of VEGF and IL-6R using HA–AuNP/TCZ complexes. Reproduced from ref. 59 with permission from American Chemical Society, copyright 2019.

Carrier-free self-assembled nanomedicines based on TCM exhibit distinct advantages in antimicrobial therapy through diverse and complementary mechanisms of action. For example, nanoparticles formed by the co-assembly of BBR and cinnamic acid (CINN) exert potent bactericidal effects against methicillin-resistant *Staphylococcus aureus* (MRSA) and effectively eradicate bacterial biofilms (Fig. 3a). Transcriptomic and quantitative PCR analyses further demonstrate that these nanoparticles elicit antibacterial activity through the coordinated regulation of multiple biological pathways.^41^ In addition, BER–RHE self-assembled nanostructures significantly lower the minimum bactericidal concentration and exhibited potent antibacterial activity by disrupting biofilms and damaging bacterial cellular structures while maintaining favorable biocompatibility.^42^

Similarly, BA and sanguinarine (SAN) elf-assembled hydrogels exhibit synergistic antibacterial activity against MRSA, suppress bacterial virulence factor expression, attenuate inflammation, and promote the healing of infected skin wounds.^43^ Beyond carrier-free small-molecule assemblies, TCM-based materials have also been applied in the green synthesis of metal nanoparticles. For example, decoctions or residues from herbs such as honeysuckle and astragalus serve as reducing and stabilizing agents in the synthesis of silver nanoparticles (TCM-derived AgNPs) (Fig. 3b). These nanoparticles demonstrate significant inhibitory effects against *Staphylococcus aureus* and *Pseudomonas aeruginosa*, and provide effective pathogen control in plant infection models, highlighting the potential of TCM-metal nanoparticle hybrid platforms.^44^

These studies indicate that self-assembled TCM nanostructures offer significant advantages in antimicrobial therapy through multi-target synergistic mechanisms. By improving drug delivery efficiency, enhancing bacterial membrane penetration, disrupting persistent biofilms, and modulating the immune microenvironment, these systems provide a promising foundation for the development of safe and effective antimicrobial therapies. However, current research remains heavily focused on *Staphylococcus aureus*, with relatively limited investigation of other clinically important pathogens such as *Pseudomonas aeruginosa*.

### 3.2 Anti-inflammatory applications

Carrier-free nanomedicines derived from TCM show substantial promise in the treatment of inflammatory diseases, with particularly well-developed evidence in RA and ulcerative colitis (UC).

#### 3.2.1 Rheumatoid arthritis

RA is a chronic, systemic autoimmune disorder characterized by persistent synovial inflammation, symmetrical polyarticular involvement, and progressive joint destruction.^45,46^ Its pathogenesis involves dysregulated immune activation, including excessive T- and B-cell responses, macrophage infiltration, and the overproduction of proinflammatory cytokines. These processes drive synovial hyperplasia, pannus formation, and subsequent cartilage and bone erosion.^47,48^ Without effective disease control, RA can lead to joint deformity and functional impairment, often accompanied by extra-articular complications such as cardiovascular disease and osteoporosis, substantially reducing quality of life.^49,50^

Current treatment strategies rely on nonsteroidal anti-inflammatory drugs (NSAIDs), glucocorticoids, disease-modifying antirheumatic drugs (DMARDs), and biologics. However, long-term use is frequently associated with adverse effects, including increased infection risk, hepatotoxicity, nephrotoxicity, and the development of drug resistance.^51^ In addition, conventional therapies often exhibit limited accumulation at inflamed joints, suboptimal targeting, and insufficient control of the complex, interconnected immune-inflammatory networks that drive disease progression. Hence, there is a need for advanced drug delivery systems capable of achieving efficient joint targeting while integrating anti-inflammatory and immunomodulatory effects.

Self-assembled nanomedicines based on TCM-derived compounds offer several advantages such as enhanced targeting, synergistic therapeutic effects, and reduced systemic toxicity. For example, following modification with hyaluronic acid (HA), the luteolin–iron coordination self-assembled nanoparticles exploit the natural affinity of HA for the CD44 receptor, which is highly expressed on the surface of various tumor cells, thereby achieving active targeting. This receptor-mediated endocytosis significantly enhances nanoparticle accumulation and cellular uptake at the tumor site. Combined with the improved stability conferred by HA modification, this carrier-free nanoplatform exhibits excellent targeted delivery performance.^52^ More recently, luteolin and glycyrrhetinic acid were reported to co-assemble into carrier-free rod-shaped nanoparticles that enhanced tumor accumulation, induced cell-cycle arrest through complementary molecular mechanisms, and significantly improved antitumor efficacy in liver cancer models while exhibiting minimal systemic toxicity.^53^ Another approach utilizes the heightened glycolytic activity observed in inflamed RA tissues. In this strategy, the glycolysis inhibitor 2-deoxy-d-glucose (2-DG) is co-assembled with the anti-inflammatory agent curcumin to form a carrier-free nanomedicine. This system eliminates the need for exogenous carriers, thereby reducing potential toxicity. Through the affinity of 2-DG for glucose transporters that are overexpressed on inflammatory cells, the nanostructures preferentially accumulate in inflamed joints. By suppressing excessive glycolysis while simultaneously exerting anti-inflammatory effects, this platform enables coordinated metabolic and immune modulation, leading to significant improvement in arthritis symptoms in RA models.^51^

To address the limitations of conventional nanocarriers, such as low drug loading and excipient-related safety concerns, Liu and colleagues developed a GBA_2_ linked *via* an ester bond. This molecule undergoes spontaneous self-assembly into carrier-free nanoparticles (GBA_2_/NPs) through π–π stacking and related intermolecular interactions, achieving drug loading as high as 85.78% along with excellent stability. These nanoparticles preferentially accumulate in inflamed joints *via* the ELVIS (extravasation through leaky vasculature and subsequent inflammatory cell-mediated sequestration) effect, and are efficiently internalized by macrophages and osteoclasts. This leads to apoptosis of pathogenic cells, thereby alleviating arthritis symptoms while minimizing systemic toxicity.^29^

#### 3.2.2 Ulcerative colitis

UC is a chronic inflammatory disorder of the colonic mucosa with an incompletely understood etiology. Oral administration remains the most practical route of treatment; however, conventional therapies often fail to achieve effective drug delivery to the colon due to degradation in the acidic gastric environment, enzymatic breakdown in the gastrointestinal tract, and limited targeting efficiency.^54^ Nanomedicines derived from TCM offer new opportunities for colon-targeted delivery. Several design strategies have been developed to enhance targeting specificity. pH-responsive systems are engineered to remain stable under gastric conditions while undergoing disassembly and drug release in the near-neutral pH environment of the colon (pH 6.8–7.4).^55^ Enzyme-responsive approaches exploit enzymes such as azoreductases produced by the abundant anaerobic microbiota in the colon, enabling site-specific cleavage of chemical bonds within nanoparticle structures or prodrugs, thereby improving drug release at inflamed sites (Fig. 3c).^56^ In addition, combining passive and active targeting strategies further enhances therapeutic efficacy. The EPR effect in inflamed intestinal mucosa facilitates preferential nanoparticle accumulation at lesion sites. Concurrently, surface modification with ligands targeting receptors overexpressed on inflammatory cells, such as CD44 and CD98, enables active targeting.^57,58^ For example, a BBR–tannic acid (TA)/HA nanosystem achieved 14.5% drug accumulation at lesion sites *via* CD44-mediated targeting and reduced the disease activity index (DAI) by 60%.^58^ HA-modified gold nanoparticles (AuNPs) conjugated with tocilizumab (TCZ) enable dual targeting of vascular endothelial growth factor (VEGF) and interleukin-6 receptor (IL-6R), thereby enhancing anti-inflammatory efficacy in RA (Fig. 3d).^59^ Similarly, curcumin-based hybrid nanostructures incorporating azoreductase-responsive linkages and catechol-modified TPGS significantly alleviated dextran sulfate sodium (DSS)-induced colitis in mice, promoted mucosal repair, and modulated gut microbiota and TLR4/MyD88/NF-κB signaling pathways.^60^

Despite these promising findings, most studies remain at the preclinical stage, with no registered Phase II or III clinical trials in RA or UC reported to date.^13,61^ Moreover, studies specifically focused on fully carrier-free nanomedicines for UC remain limited. Nonetheless, research on colon-targeted nanosystems based on TCM-derived active compounds has yielded encouraging results. For instance, studies in a model of ulcerative colitis have demonstrated that naturally occurring polyphenol–nobiletin self-assembled nanoparticles with a uniform particle size of approximately 250 nm can maximize their anti-inflammatory and antioxidant activities, effectively alleviating intestinal inflammation and restoring the mucosal barrier. These findings provide an important reference for optimizing the design parameters of nanomedicines,^62^ providing valuable guidance for optimizing nanomedicine design parameters. BBR self-assembled nanoparticles have been shown to significantly reduce disease severity in a DSS-induced colitis model by modulating gut microbiota composition and attenuating pro-inflammatory cytokine expression while improving mucosal barrier integrity.^63^ In another study, an optimized TA-BBR self-assembled system coated with permeable HA enabled targeted colonic delivery through HA–CD44 interactions.^58^ This platform was comprehensively characterized, and its biodistribution, therapeutic efficacy, and underlying mechanisms were systematically evaluated in a DSS-induced colitis model.

### 3.3 Tumor-targeted therapy

In recent years, carrier-free self-assembled nanodelivery systems based on active components of TCM have attracted increasing attention in cancer therapy. These systems use natural compounds or their derivatives as building blocks to form well-defined nanostructures through relatively simple and environmentally friendly processes. By eliminating synthetic carriers, they reduce biosafety concerns while retaining the intrinsic biological activity of the constituent molecules. Owing to their tumor-targeting capability and potential to induce immunogenic cell death, such as pyroptosis and ferroptosis, these systems show considerable promise in the treatment of solid tumors, including hepatocellular carcinoma (HCC) and breast cancer. The following sections summarize recent advances in these areas.

#### 3.3.1 Hepatocellular carcinoma

Carrier-free self-assembled nanomedicines derived from TCM exhibit notable advantages in the treatment of HCC, including enhanced efficacy and reduced systemic toxicity. Through rational design and modification of natural compounds, these systems enable synergistic effects spanning drug delivery, tumor suppression, and immune modulation. For example, UA and epigallocatechin gallate (EGCG) have been co-assembled into a core–shell nanostructure using a green synthesis approach, followed by modification with an EpCAM-targeting aptamer for active tumor recognition.^64^ This nanoplatform demonstrates high stability, pH responsiveness, and deep tumor penetration. In another study, berberine and artemisinin can co-assemble into nanoparticles without carriers, resulting in complementary antitumor effects and enhanced cytotoxicity in HCC models relative to monotherapy, attributed to synchronized ROS generation and apoptosis induction.^65^ The resulting system enables sustained drug release and improved therapeutic efficacy at lower doses.

Celastrol (CE), a bioactive compound from TCM, has also been conjugated with galactose (Gal) to form carrier-free CE-Gal nanoparticles *via* nanoprecipitation. The galactose moiety enables active targeting by binding to asialoglycoprotein receptors on HCC cells. In combination with the EPR effect, this promotes selective accumulation within tumor tissue. Following cellular uptake, CE induces the generation of reactive oxygen species (ROS), downregulates glutathione peroxidase 4 (GPX4), and upregulates cyclooxygenase-2 (COX-2), ultimately triggering ferroptosis in HCC cells.^66^ This system improves the solubility and targeting efficiency of CE while reducing systemic toxicity, highlighting the potential of single-component TCM-derived nanomedicines for ferroptosis-based cancer therapy.

These studies demonstrate that self-assembled nanostructures derived from TCM active components can overcome key limitations of conventional chemotherapy through multi-component synergy, targeted delivery, and immunometabolic regulation. By eliminating carrier-related toxicity and leveraging the multi-target properties of natural compounds, these systems offer promising strategies for improving the safety and efficacy of hepatocellular carcinoma treatment.

#### 3.3.2 Breast cancer

Recent breast cancer research increasingly emphasizes synergistic strategies that combine immune activation with the induction of programmed cell death. Nanoscale systems constructed *via* molecular self-assembly of active components from TCM provide innovative approaches for integrating chemotherapy, photodynamic therapy (PDT), and immune microenvironment modulation.

For example, researchers developed a co-delivery nanomedicine system (PPDC) incorporating dihydroartemisinin (DHA) and chlorin e6 (Ce6) to enhance PDT efficacy in breast cancer. The PPDC nanoparticles, based on polyethylene glycol-poly(ε-caprolactone) (PEG-PCL), encapsulate both agents to improve solubility, tumor targeting, and therapeutic performance. Experimental results demonstrate that PPDC efficiently delivers DHA and Ce6 to tumor cells, promoting ROS generation and enhancing apoptosis. These findings highlight the potential of PPDC as an effective nanomedicine platform for breast cancer treatment.^67^

BA, the primary active component of *Coptis chinensis* and *Scutellaria baicalensis*, also demonstrates significant translational potential in breast cancer therapy. Notably, its nanodelivery systems have been shown to markedly enhance antitumor efficacy.^68^ BA nanoparticles modulate TGF-β signaling, which influences lncRNA-MALAT1 expression and subsequently upregulates miR-200c. miR-200c directly targets and suppresses the epithelial–mesenchymal transition (EMT) transcription factors ZEB1/2, thereby reversing EMT, restoring the epithelial phenotype, and inhibiting cancer cell migration and invasion.^69,70^ Furthermore, the FA-ZIF-8/BA nanoparticle system utilizes the high drug-loading capacity of the metal–organic framework ZIF-8 and achieves active targeting *via* folate modification. This design significantly increases drug accumulation at tumor sites, offering a promising strategy for targeted breast cancer therapy.^71^ In addition, a novel Zn(ii)–Schiff base complex (Zn–MTDH) was rationally designed through coordination chemistry to achieve lysosomal targeting and high-contrast fluorescence imaging with a quantum yield of 63.7% in 4T1 breast cancer cells. Upon loading with camptothecin (CPT), CPT@Zn-MTDH exhibited pH-dependent drug release kinetics and enhanced cytotoxicity compared to free CPT, establishing a versatile track and treat theranostic platform for lysosome-targeted cancer therapy.^72^ Although direct evidence for BAI's fully carrier-free self-assembly or ferroptosis induction is limited, its unique chemical structure and multi-target pharmacological profile make it a strong candidate for developing carrier-free nanomedicines for breast cancer.

#### 3.3.3 Melanoma

Melanoma is the most malignant and aggressive type of skin cancer, characterized by a high metastatic rate and an immunosuppressive tumor microenvironment. Conventional treatments, including surgery, chemotherapy, and immune checkpoint inhibitors, exhibit limited response rates in patients with advanced or metastatic disease, resulting in an extremely poor prognosis.^73^ In recent years, self-assembled nanoplatforms based on the synergy of phototherapy and immunomodulation have provided new strategies for the theranostics of melanoma. By co-assembling near-infrared photosensitizers and immunomodulators within nano-delivery systems, it is possible to achieve the organic integration of precise tumor imaging, localized thermal ablation, and systemic immune activation, thereby effectively inhibiting the growth of both primary tumors and metastatic lesions.^74^

Sun *et al.* constructed acedan nanoparticles (INEs) co-loaded with the photosensitizer IR251 and the IDO inhibitor NLG919. Upon 808 nm laser irradiation, INEs induced immunogenic cell death (ICD) of tumor cells through photothermal/photodynamic effects, leading to the release of CRT and HMGB1, which promoted dendritic cell maturation and CD8^+^ T cell infiltration. In a B16 bilateral tumor-bearing mouse model, INEs combined with laser treatment not only significantly suppressed the primary tumor but also effectively inhibited distant metastatic tumors through systemic immune activation.^75^ Furthermore, Yang *et al.* designed a nanoplatform (IRM) co-loaded with the NIR-II fluorescent dye IR-817 and the STING agonist MSA-2.^76^ This platform enabled NIR-II fluorescence imaging-guided photothermal therapy, achieving a photothermal conversion efficiency of 52.79%. Laser irradiation triggered the on-demand release of MSA-2, activating the STING pathway and synergizing with photothermal-induced ICD to remodel the tumor immune microenvironment. In the B16 model, IRM combined with laser treatment significantly inhibited both primary and distant tumors; however, the distant tumor suppression effect was completely abolished in STING knockout mice, confirming that the STING pathway is critical for systemic antitumor immunity. Moreover, hypoxia in the tumor microenvironment represents a critical barrier to effective melanoma immunotherapy by suppressing T cell infiltration and function. Oxygen-supplied nanomaterials (OSNs) alleviate hypoxia, restore T cell activity, and enhance chemokine-mediated T cell recruitment, thereby synergizing with immune checkpoint inhibitors and CAR-T cell therapies to overcome hypoxia-driven immune suppression. Key OSN systems, including perfluorocarbon-based and catalytic nanoparticles, have demonstrated transformative potential to enhance T cell-mediated anti-tumor immunity and advance the frontier of melanoma immunotherapy.^77^

The aforementioned studies demonstrate that adaptive nanoplatforms based on the co-assembly of photosensitizers and immunomodulators, integrating NIR-II imaging-guided photothermal/photodynamic therapy, can effectively overcome the immunosuppressive microenvironment of melanoma and induce durable systemic antitumor immune responses. This approach advances the precision theranostics of advanced metastatic melanoma. Future research should focus on further optimizing the targeted delivery efficiency and biosafety evaluation of nanoformulations to facilitate their clinical translation.

## 4. Core challenges and strategies for clinical translation

### 4.1 Comparison with conventional nanocarriers

As noted in Section 2.2, although advanced preparation technologies enable efficient and versatile self-assembly, they also introduce challenges related to process sensitivity and reproducibility. Unlike conventional nanocarriers, carrier-free self-assembled systems derived from TCM rely entirely on weak, reversible noncovalent interactions (*e.g.*, π–π stacking, hydrogen bonding, and electrostatic forces), which fundamentally distinguishes their structural stability and *in vivo* behavior. Carrier-free nanomedicines may present several limitations when compared with conventional nanocarrier systems such as liposomes and polymeric nanoparticles. One of the primary disadvantages is reduced structural stability, as multi-component TCM assemblies are particularly susceptible to environmental perturbations (*e.g.*, pH, ionic strength, and serum protein interactions), leading to aggregation, premature disassembly, or structural rearrangement during storage and circulation. In contrast, carrier-based systems typically provide a more stable and controllable structural framework. In addition, carrier-free systems often exhibit limited control over pharmacokinetics and drug release profiles, as the absence of an engineered carrier restricts opportunities for sustained or stimuli-responsive release design. Although high drug loading is a key advantage, it may also lead to rapid drug release and off-target effects if not properly controlled. Furthermore, the inherent chemical heterogeneity of TCM-derived components introduces additional complexity in multi-component co-assembly, where subtle variations in composition or molecular ratio can significantly alter nanostructure formation, biological activity, and safety profiles. Batch-to-batch variability and challenges in reproducibility remain significant barriers for large-scale production.

However, carrier-based nanomedicines may remain preferable in several scenarios. First, when high structural stability and long circulation time are required, well-established platforms such as liposomes or polymeric nanoparticles provide more reliable performance. Second, for applications requiring precise control over drug release kinetics, including sustained or stimuli-responsive delivery, engineered carriers offer greater flexibility. Third, in clinical settings, regulatory familiarity and established safety profiles of carrier-based systems may facilitate translation. Finally, for drugs with poor self-assembly capability or requiring protection from degradation, carrier-based formulations remain advantageous.

### 4.2 Key technical barriers and safety considerations

#### 4.2.1 Limited process control

Batch-to-batch variability remains a major barrier to the industrialization of carrier-free nanomedicines. This issue is particularly pronounced in TCM-based systems due to the variability in source materials, extraction processes, and compositional complexity of active constituents. For example, although the ice-templating method allows particle size control within the range of 50–200 nm,^30^ the polydispersity index (PDI) frequently exceeds 0.3 across different production batches. In addition, equipment wear may introduce contaminants, such as aluminum ions, further complicating the standardization of manufacturing processes required for clinical-grade production. More importantly, for multi-component self-assembled systems, small deviations in component ratio or solvent conditions can lead to significant shifts in intermolecular interactions, resulting in inconsistent nanostructures and therapeutic outcomes.

#### 4.2.2 Complexity of *in vivo* behavior

The *in vivo* behavior of TCM-derived nanomedicines is highly complex, with safety profiles influenced by both the intrinsic toxicity of active compounds and their metabolic and clearance characteristics. In carrier-free systems, where the nanostructure itself is composed entirely of bioactive molecules, pharmacokinetics and toxicity are tightly coupled to the dynamic stability of the self-assembled structure. For instance, tripterygium glycosides (TGT) exhibit potent antitumor and immunosuppressive effects but are also associated with significant multi-organ toxicity, including adverse effects on the liver, kidneys, heart, and reproductive system.^78–80^ In a self-assembled context, structural disassembly or premature drug release may further amplify systemic exposure and toxicity. Similarly, PEGylated vitamin E nanoparticles demonstrate prolonged circulation time; however, they may also trigger accelerated blood clearance upon repeated administration, with clearance rates increasing by up to 300%, thereby raising potential immunogenicity concerns.^81^

#### 4.2.3 Lag in regulatory frameworks

The regulatory landscape for carrier-free self-assembled nanomedicines remains underdeveloped, reflecting the complex nature of these multi-component systems, particularly for multi-component TCM systems with complex and dynamic supramolecular architectures. For example, the National Medical Products Administration (NMPA, China) currently lacks a dedicated regulatory category for such formulations, and standardized quality control criteria, especially those addressing self-assembly stability, component ratio consistency, and structure–function relationships, are not established.^82,83^ Internationally, frameworks from the U.S. Food and Drug Administration (FDA) and European Medicines Agency (EMA) provide general guidance for nanomedicines but do not specifically address carrier-free, composition-dependent assemblies. Key quality attributes critical for these systems include particle size distribution, PDI, drug loading efficiency, surface charge, and structural stability in biologically relevant media (*e.g.*, plasma protein-containing environments). Batch-to-batch reproducibility can be validated using advanced characterization techniques (*e.g.*, dynamic light scattering, high-performance liquid chromatography, and cryo-TEM) combined with statistical process control.^84^ For multi-component systems, orthogonal analytical approaches are particularly important to simultaneously monitor composition, nanostructure integrity, and functional performance.

### 4.3 Collaborative innovation strategies and practical advances

#### 4.3.1 Precision manufacturing approaches

Microfluidic technologies enable precise control over nanoparticle formation by regulating fluid flow and mixing within microscale channels. This approach can achieve a coefficient of variation (CV) in particle size of less than 5%, ensuring high structural uniformity and monodispersity. Importantly, such precise control is especially valuable for TCM-derived multi-component systems, where reproducibility depends on maintaining consistent intermolecular interaction conditions during assembly. Furthermore, integrating microfluidics with molecular dynamics simulations to predict self-assembly behavior can substantially reduce reagent consumption and decrease trial-and-error costs by more than 80%.^85,86^

#### 4.3.2 Strategies for enhancing stability

Optimized freeze–drying (lyophilization) techniques play a critical role in maintaining nanoparticle stability. Rapid cooling (*e.g.*, −80 °C min^−1^) helps preserve the original particle size distribution, while the addition of cryoprotectants such as trehalose reduces freeze-induced stress. However, for carrier-free systems, the introduction of stabilizing agents must be carefully controlled to avoid altering the intrinsic intermolecular interactions that drive self-assembly, thereby preserving the “carrier-free” nature of the formulation. Careful control of vacuum drying conditions, including maintaining residual moisture levels ≤1%, can improve the stability of solid formulations by more than threefold. These strategies help preserve nanoparticle integrity and prevent issues such as aggregation, drug leakage, and hydrolytic degradation during long-term storage.^87^

#### 4.3.3 Smart delivery system design

Smart delivery system design remains an important challenge for the clinical translation of carrier-free self-assembled nanomedicines. Although considerable progress has been made in developing targeted delivery platforms, many current approaches rely on complex functional modifications that may increase manufacturing complexity, reduce reproducibility, and complicate regulatory approval. For example, hyaluronic acid-modified erythrocyte membrane-coated nanoparticles, formed by the self-assembly of celastrol and bilirubin, employ biomimetic camouflage technology to effectively evade immune clearance and prolong circulation time. Simultaneously, through active targeting of inflamed joints, inhibition of the STING pathway, and scavenging of reactive oxygen and nitrogen species, these nanoparticles alleviate rheumatoid arthritis while reducing drug-induced hepatotoxicity.^88^ Furthermore, self-targeting design can be achieved by exploiting the intrinsic targeting moieties of drug molecules. For instance, methotrexate, utilizing its own folate moiety, can be used to construct a carrier-free nanosystem, which enables active targeted delivery while simultaneously enhancing antitumor synergy and reducing toxicity.^89^ Although these studies demonstrate the feasibility of smart delivery strategies, further investigation is required to evaluate their long-term safety, manufacturing scalability, and translational potential.

However, such modifications should be carefully distinguished from strictly defined carrier-free systems, as they introduce exogenous materials that may alter the original self-assembly behavior and pharmacokinetics of TCM-derived nanostructures. Tumor microenvironment-responsive systems provide another level of control. These platforms often incorporate pH- or charge-responsive self-assembling peptides that undergo physicochemical changes under acidic tumor conditions. For instance, the zeta potential of GC-SPIO nanoparticles increases from +0.3 mV at pH 7.4 to +8.2 mV at pH 6.15, facilitating enhanced interaction with tumor cells.^90^ In addition, the integration of TCM formulation theory, specifically the “sovereign–minister–adjuvant–messenger” framework, provides a unique strategy for rationally designing multi-component self-assembled systems, enabling coordinated modulation of efficacy and toxicity through controlled component interactions.^91^ Nevertheless, the responsiveness of these systems may vary across different pathological microenvironments.

#### 4.3.4 Integration of formulation technologies

Nanocrystal engineering offers an effective strategy to improve the dissolution rate and bioavailability of poorly soluble TCM-derived compounds, such as silymarin, by controlling particle size and surface characteristics, thereby facilitating oral delivery.^92^ Pharmacokinetic/pharmacodynamic (PK/PD) studies also play a critical role in formulation development. For example, systematic evaluation of tissue distribution, such as liver and spleen targeting of *Angelica sinensis* polysaccharides, provides key evidence for optimizing formulation design and dosing strategies, thereby strengthening preclinical validation.^93^ For carrier-free systems, such studies are particularly important to correlate dynamic structural stability with *in vivo* distribution and therapeutic performance. Moreover, integrating formulation development with Quality by Design (QbD) principles, through the identification of critical material attributes and process parameters and the establishment of defined design spaces, supports consistent product quality and robust manufacturing control.

#### 4.3.5 Establishment of a multidimensional evaluation framework

A comprehensive evaluation of carrier-free nanomedicines requires a multidimensional and multilevel assessment framework. Future efforts should focus on developing organ-specific PK/PD models, for example, in the case of a model for metabolic dysfunction-associated steatotic liver disease (MASLD), the model should encompass key pathophysiological processes, including intrahepatocellular lipid accumulation, regulation of genes involved in lipid synthesis and oxidation, hepatocellular injury, and the reversal of hepatic steatosis.^94^ At the same time, there is a need for advanced high-throughput analytical platforms capable of resolving complex multi-component interactions. Such platforms would support systematic investigation of co-assembly systems involving three or more agents, enabling reliable identification of synergistic effects and facilitating the development of rational combination therapies.^95^ This is particularly critical for TCM-based systems involving three or more co-assembled components, where emergent properties cannot be predicted from individual constituents alone. From a regulatory science perspective, greater alignment with international frameworks, such as ICH-Q14, will be essential. Promoting the application of QbD principles and establishing review standards tailored to the composition-dependent and dynamically assembled nature will help accelerate clinical translation.

## 5. Conclusion and future perspectives

Carrier-free self-assembled nanomedicines derived from TCM have demonstrated distinct physicochemical and functional advantages across the studies reviewed. Most reported systems are constructed from alkaloids (*e.g.*, berberine), flavonoids (*e.g.*, baicalin and EGCG), and triterpenes (*e.g.*, ursolic acid and celastrol), frequently forming alkaloid–polyphenol or terpenoid-based co-assemblies. These systems are predominantly driven by π–π stacking, hydrogen bonding, and electrostatic interactions, resulting in well-defined nanostructures such as nanoparticles, hydrogels, and core–shell structures. Across antibacterial applications, multiple studies demonstrate effective biofilm disruption, bacterial membrane damage, and efflux pump inhibition, as observed in berberine-based co-assemblies. In anti-inflammatory and metabolic disease models, co-assembled systems (*e.g.*, curcumin–2-deoxy-d-glucose) regulate glycolysis, immune responses, and gut microbiota. In tumor models, synergistic mechanisms including ROS generation, ferroptosis induction, EMT inhibition, and immune activation are consistently reported. In addition, several studies incorporate targeting strategies such as CD44-mediated colon targeting, glucose transporter affinity, EpCAM aptamer recognition, and folate receptor modification, enhancing site-specific accumulation and therapeutic efficacy.

Despite these encouraging advances, the clinical translation of carrier-free TCM nanomedicines will depend on addressing several fundamental challenges. Rather than pursuing numerous parallel developments, future research should prioritize three key areas that are both scientifically important and achievable in the near term.

First, establishing predictive design principles for molecular self-assembly should be a primary research priority. Current formulation strategies remain largely empirical, with the selection of component combinations and preparation conditions relying heavily on trial-and-error experimentation. Future studies should integrate molecular dynamics simulations, computational chemistry, and machine learning with systematic experimental validation to establish quantitative relationships between molecular structure, intermolecular interactions, assembly morphology, and biological performance. A particularly important hypothesis is that molecular descriptors, including aromaticity, hydrogen-bonding capacity, hydrophobicity, and molecular flexibility, can be used to predict self-assembly behavior and guide the rational design of carrier-free nanosystems.

Second, improving the *in vivo* stability and translational performance of carrier-free nanomedicines should receive greater attention. Although many self-assembled systems exhibit excellent stability under laboratory conditions, they may undergo partial disassembly following plasma dilution, protein corona formation, competitive molecular interactions, or enzymatic degradation after systemic administration. Future investigations should systematically correlate assembly architecture, physicochemical properties, pharmacokinetics, biodistribution, and therapeutic efficacy across different disease models.

Third, standardized evaluation frameworks are urgently needed to facilitate reproducibility and clinical translation. Future studies should move beyond demonstrating therapeutic efficacy alone and adopt harmonized protocols for physicochemical characterization, *in vitro* and *in vivo* stability assessment, pharmacokinetic evaluation, biodistribution analysis, and long-term safety testing. The implementation of QbD principles, together with standardized analytical methodologies, will improve manufacturing consistency and provide the high-quality datasets required for regulatory evaluation and AI-assisted formulation optimization.

In summary, carrier-free self-assembled nanomedicines derived from TCM are positioned at the intersection of traditional medical knowledge and modern nanotechnology. Continued progress in these prioritized areas is expected to accelerate the development of safe, reproducible, and precisely engineered nanomedicines capable of addressing complex diseases and supporting future clinical translation.

## Author contributions

Shuhan Yan: writing – original draft, data curation, investigation. Enyao He: writing – original draft, methodology. Meiting Chen: data curation, Xingbo Wang: conceptualization, Changzhen Sun: writing – review and editing, Fund acquisition.

## Conflicts of interest

There are no conflicts to declare.

## Data Availability

No new data were created or analyzed in this study.
